# Substantial Diversity in Cocirculating Omicron Lineages in Hospital Setting, Porto Alegre, Brazil

**DOI:** 10.3201/eid2912.230880

**Published:** 2023-12

**Authors:** Tiago F. Andreis, Vlademir V. Cantarelli, Marcelo B. da Silva, Mateus S. Helfer, Flávia R. Brust, GAIHN-HAI Team, Alexandre P. Zavascki

**Affiliations:** Hospital Moinhos de Vento, Porto Alegre, Brazil (T.F. Andreis, M.S. Helfer, A.P. Zavascki);; Universidade Federal de Ciências da Saúde de Porto Alegre, Porto Alegre (V.V. Cantarelli);; Unimed Vale dos Sinos, Novo Hamburgo, Brazil (M.B. da Silva);; Universidade do Vale do Rio dos Sinos, São Leopoldo, Brazil (M.B. da Silva);; Health Security Partners, Washington, DC, USA (F.R. Brust);; Universidade Federal do Rio Grande do Sul, Porto Alegre (A.P. Zavascki)

**Keywords:** SARS-CoV-2, Omicron, BE.9, Brazil, COVID-19, coronavirus disease, severe acute respiratory syndrome coronavirus 2, viruses, respiratory infections, zoonoses, vaccine-preventable diseases

## Abstract

We describe substantial variant diversity among 23 detected SARS-CoV-2 Omicron lineage viruses cocirculating among healthcare workers and inpatients (272 sequenced samples) from Porto Alegre, Brazil, during November 2022–January 2023. BQ.1 and related lineages (61.4%) were most common, followed by BE.9 (19.1%), first described in November 2022 in the Amazon region.

When SARS-CoV-2 variants of concern were first described, the epidemiologic situation was characterized by sequential waves of Alpha, Beta, Gamma, and Delta variants, with relatively few other variants cocirculating with the dominant variant of concern of each wave (*1*). The epidemiologic situation shifted with the emergence of the Omicron variant (B.1.1.529) in November 2021 (*2*). Distinct Omicron lineages rapidly emerged, causing successive, relatively narrow waves of infection associated with novel lineages that had pronounced immune escape and increased transmissibility (*3*).

The Global Action in Healthcare Network–Healthcare-associated Infection (GAIHN-HAI) module is a multinational network of healthcare facilities and laboratories developed by the Division of Healthcare Quality Promotion, National Center for Emerging and Zoonotic Infectious Diseases, US Centers for Disease Control and Prevention (Atlanta, GA, USA), to address emerging infectious disease threats in healthcare settings. The network began genomic surveillance of SARS-CoV-2 lineages affecting healthcare workers (HCWs) and inpatients in Brazil in November 2022. We present initial findings from a tertiary-care COVID-19 reference hospital in Porto Alegre, the capital of the southernmost state of Brazil.

We conducted this surveillance study based on data from Hospital Moinhos de Vento, the first facility to join the Brazil GAIHN-HAI network. We obtained demographics and exposure risk factors by participant interview and invited into the study all HCWs and inpatients ≥18 years of age with COVID-19 diagnosed by real-time reverse transcription PCR that had a cycle threshold <30 in any probe. We performed whole-genome sequencing (Appendix) and submitted all viral genome sequences to GISAID (http://www.gisaid.org) (Appendix Table 1) (*4*).

During November 2022–January 2023, we collected 552 deduplicated SARS-CoV-2 real-time reverse transcription PCR–positive specimens (360 [65.2%] HCWs, 192 [34.8%] inpatients). Of the 552 specimens, we excluded 124 (22.5%) because cycle threshold was >30 and 156 (28.3%) because we were unable to obtain consent for, resulting in 272 (49.3%) samples sequenced and available for analysis. Analyzed samples consisted of 182 (66.9%) samples from HCWs and 90 (33.1%) from inpatients.

We identified 23 distinct lineages, all belonging to the Omicron variant (Table; Appendix Figure). BQ.1 and related lineages were most prevalent (61.4%), followed by BE.9 (19.1%) and others (19.5%). We detected BE.9 first, in epidemiologic week 45 of 2022, and that lineage remained cocirculating with BQ.1 in a subdominant proportion of cases throughout the study period (Appendix Figure). We also noted genetic relatedness of other Omicron lineages (Figure).

**Table:** Identification of Omicron SARS-CoV-2 lineages among healthcare workers and inpatients in Porto Alegre, southern Brazil, November 2022–January 2023

Group	Lineage	No. cases	% Cases	Cumulative %
BQ.1, n = 167 (61.4%)	BQ.1.1	140	51.5	51.5
	BQ.1.1.18	14	5.1	56.6
	BQ.1.3	3	1.1	57.7
	BQ.1	3	1.1	58.8
	BQ.1.1.23	2	0.7	59.6
	BQ.1.1.15	1	0.4	59.9
	BQ.1.1.17	1	0.4	60.3
	BQ.1.1.22	1	0.4	60.7
	BQ.1.1.24	1	0.4	61.0
	BQ.1.1.4	1	0.4	61.4
BE.9, n = 52 (19.1%)	BE.9	52	19.1	80.5
BA.5, n = 15 (5.5%)	BA.5.3.1	6	2.2	82.7
	BA.5	4	1.5	84.2
	BA.5.2.1	3	1.1	85.3
	BA.5.1.27	2	0.7	86.0
Other lineages, n = 38 (14%)	BE.10	14	5.1	91.2
	CK.1	11	4.0	95.2
	DL.1	3	1.1	96.3
	XBB.1	3	1.1	97.4
	XBB.1.5	1	0.4	97.8
	BA.4.6	3	1.1	98.9
	BN.1.3.1	2	0.7	99.6
	BN.1.5	1	0.4	100.0
Total		272	100.0	100.0

**Figure:**
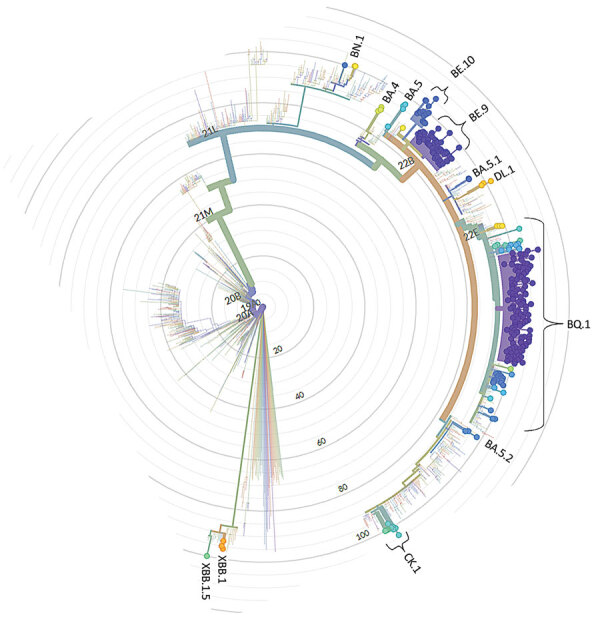
Phylogenetic radial tree showing placement of 272 SARS-CoV-2 sequenced samples from healthcare workers and inpatients in Porto Alegre, southern Brazil, November 2022–January 2023. Data as of February 25, 2023.

We noted no difference in the distribution of lineages between HCWs and inpatients. Compared with other lineages, BE.9 was more common in female patients (p = 0.027), younger patients (p = 0.017), and patients reporting previous contact with a person infected with SARS-CoV-2 (p≤0.001). We noted vaccination status and report of a previous infection were similar among participants, regardless of lineage (Appendix Table 2).

Our findings from a select population of HCWs and inpatients from a hospital in southern Brazil revealed the cocirculation of 23 Omicron lineages over a relatively short (11-week) period. Although we observed BQ.1-related lineages most frequently, consistent with this lineage’s recent predominance both globally (*5*) and in Brazil (*3*), the number of subvariants we observed represents a departure from the observed serial dominance common with earlier variants of concern (*1*,*6*). Generalizability of our findings is limited, but our observations are consistent with other global findings suggesting that Omicron has diversified to include multiple lineages, with adequate fitness allowing them to cocirculate among humans (*2*,*3*,*7*,*8*). One proposed explanation is that population-level variation in vaccination and previous infection has led to heterogeneous immunologic background, leading to cocirculation of distinct lineages with varying proficiency for natural and induced immunogenic escape (*3*,*7*). However, recent research suggests that immune imprinting induces Omicron receptor-binding protein mutation convergence (*9*).

One unexpected finding of this study was the relatively high proportion of BE.9 lineages we observed. BE.9 was first described in November 2022 in the northern Brazil state of Amazonas (*7*) and is characterized by a large, 244-nt deletion in the open reading frame (ORF) 7a gene at the position 27508–27751 and by mutations at spike:K444T, spike:N460K, spike:Y144del, ORF1a:V84del, and ORF1a:M85del (https://github.com/cov-lineages/pango-designation/issues/1302). Since its initial description in Brazil, BE.9 has been reported in other countries, but only a small portion of those identified sequences have been submitted to GISAID (*4*). As for BE.9, most BE.10 cases (≈75%) reported in GISAID are from Brazil (*4*). In contrast to other countries, where the recombinant XBB.1.5 has been circulating since August 2022 (*10*), we only detected XBB.1.5 in the study population starting January 19, 2023.

In conclusion, SARS-CoV-2 genomic surveillance at a hospital in southern Brazil found substantial diversity of Omicron lineages among HCWs and inpatients. Findings are specific to this facility and not generalizable to other hospitals or the population of Brazil. As countries globally adapt their national SARS-CoV-2 testing strategies to current COVID-19 epidemiology, they should consider focusing SARS-CoV-2 genomic surveillance strategies, along with infection trend monitoring on smaller, targeted populations such as HCWs and inpatients, to identify unusual epidemiologic events, characterize unusual viral transmission chains, and guide facility-level response measures.

Appendix More information for substantial diversity in cocirculating Omicron lineages in hospital setting, Porto Alegre, Brazil.
