# Supplementary material for: Substantial Diversity in Cocirculating Omicron Lineages in Hospital Setting, Porto Alegre, Brazil
Source: Emerg Infect Dis. 2023 Dec;29(12):2583–6. doi: 10.3201/eid2912.230880 (PMC10683828; doi:10.3201/eid2912.230880)
Supplement: Appendix — More information for substantial diversity in cocirculating Omicron lineages in hospital setting, Porto Alegre, Brazil. [file 23-0880-Techapp-s1.pdf]

Article DOI: https://doi.org/10.3201/eid2912.230880

EID cannot ensure accessibility for supplementary materials supplied by authors. Readers who have difficulty accessing supplementary content should contact the authors for assistance.

Substantial Diversity in Cocirculating Omicron Lineages in Hospital Setting, Porto Alegre, Brazil

Appendix

Methods used for molecular testing and whole-genome sequencing and ethical considerations

Real-time RT-PCR

Real-time RT-PCRs were performed by using the commercial kits TaqMan 2019-nCoV Assay Kit v1 (Thermo Fisher Scientific, https://www.thermofisher.com/us), TaqCheck SARS-CoV-2 Fast PCR Assay (Thermo Fisher Scientific) or Xpert Xpress SARS-CoV-2 (Cepheid, https://www.cepheid.com/en-US).

Whole-Genome Sequencing

The viral genomic library was prepared by using the CleanPlex SARS-CoV-2 FLEX Kit and the SARS-CoV-2 Emerging Variants Panel Add-on v2 (Paragon Genomics, Inc, www.paragongenomics.com) following the manufacturer's instructions. The resulting libraries were sequenced on the Illumina MiSeq platform by using a V2 chemistry (Illumina, https://www.illumina.com).

Ethical approvals

The study was approved by the Brazilian National Health Council - Ministry of Health (CAAE: 59038722.0.1001.5330) and informed consent was obtained from all participants.

Appendix Table 1. GISAID accession number of all viral genome sequences from a study of healthcare workers and inpatients at Hospital Moinhos de Vento in Porto Alegre, southern Brazil, November 2022–January 2023

Lineage	GISAID accession number
BQ.1.1	EPI_ISL_15803844, EPI_ISL_15803846, EPI_ISL_16002026, EPI_ISL_15803851, EPI_ISL_15803853, EPI_ISL_16002028, EPI_ISL_16002021, EPI_ISL_16002008, EPI_ISL_16002025, EPI_ISL_16002022, EPI_ISL_16002017, EPI_ISL_16002027, EPI_ISL_16002019, EPI_ISL_16002014, EPI_ISL_16002012, EPI_ISL_16360410, EPI_ISL_16360411, EPI_ISL_16002015, EPI_ISL_16002024, EPI_ISL_16360412, EPI_ISL_16360413, EPI_ISL_16360415, EPI_ISL_16360416, EPI_ISL_16360418, EPI_ISL_16360419, EPI_ISL_16360420, EPI_ISL_16360421, EPI_ISL_16360422, EPI_ISL_16360423, EPI_ISL_16360425, EPI_ISL_16360429, EPI_ISL_16360383, EPI_ISL_16360430, EPI_ISL_16360432, EPI_ISL_16360434, EPI_ISL_16360435, EPI_ISL_16360436, EPI_ISL_16360395, EPI_ISL_16360428, EPI_ISL_16360399, EPI_ISL_17651884, EPI_ISL_16360437, EPI_ISL_16360438, EPI_ISL_16360439, EPI_ISL_16360440, EPI_ISL_17848718, EPI_ISL_16360403, EPI_ISL_16360385, EPI_ISL_16423445, EPI_ISL_16360404, EPI_ISL_16360386, EPI_ISL_16360387, EPI_ISL_16360389, EPI_ISL_16360405, EPI_ISL_16360408, EPI_ISL_16360393, EPI_ISL_16360394, EPI_ISL_16360409, EPI_ISL_17848770, EPI_ISL_17848775, EPI_ISL_17848758, EPI_ISL_16360398, EPI_ISL_17651886, EPI_ISL_17651885, EPI_ISL_17848790, EPI_ISL_17651894, EPI_ISL_16360362, EPI_ISL_16360366, EPI_ISL_17651887, EPI_ISL_16360363, EPI_ISL_17848761, EPI_ISL_17651889, EPI_ISL_17848762, EPI_ISL_16360365, EPI_ISL_17848722, EPI_ISL_17848724, EPI_ISL_17848765, EPI_ISL_17848774, EPI_ISL_17848764, EPI_ISL_17651891, EPI_ISL_17848768, EPI_ISL_16360373, EPI_ISL_16360369, EPI_ISL_16360370, EPI_ISL_16360372, EPI_ISL_17848793, EPI_ISL_17848769, EPI_ISL_17651892, EPI_ISL_16360374, EPI_ISL_17848776, EPI_ISL_16360371, EPI_ISL_17848794, EPI_ISL_16360375, EPI_ISL_17848771, EPI_ISL_17848772, EPI_ISL_17848777, EPI_ISL_17848773, EPI_ISL_16360377, EPI_ISL_16360381, EPI_ISL_16360376, EPI_ISL_17848778, EPI_ISL_16360380, EPI_ISL_17848779, EPI_ISL_17651893, EPI_ISL_16360382, EPI_ISL_17848780, EPI_ISL_17848791, EPI_ISL_17848785, EPI_ISL_17848786, EPI_ISL_17848792, EPI_ISL_17848726, EPI_ISL_17848730, EPI_ISL_18103841, EPI_ISL_17848729, EPI_ISL_17848727, EPI_ISL_17848789, EPI_ISL_17848731, EPI_ISL_17848736, EPI_ISL_17848734, EPI_ISL_18103818, EPI_ISL_17848746, EPI_ISL_17848737, EPI_ISL_17848742, EPI_ISL_17848741, EPI_ISL_17848744, EPI_ISL_17848738, EPI_ISL_17848757, EPI_ISL_17848739, EPI_ISL_17848740, EPI_ISL_17848748, EPI_ISL_17848751, EPI_ISL_17848752, EPI_ISL_17848754, EPI_ISL_17848800, EPI_ISL_17848753, EPI_ISL_17848803, EPI_ISL_17848759, EPI_ISL_17848797, EPI_ISL_17848805, EPI_ISL_17848798
BQ.1.1.18	EPI_ISL_15803847, EPI_ISL_16360414, EPI_ISL_16360396, EPI_ISL_16360401, EPI_ISL_16360388, EPI_ISL_16360407, EPI_ISL_16360391, EPI_ISL_17651890, EPI_ISL_17848743, EPI_ISL_17848766, EPI_ISL_17848781, EPI_ISL_16360379, EPI_ISL_17848787, EPI_ISL_17848750
BQ.1.3	EPI_ISL_16360431, EPI_ISL_16360402, EPI_ISL_16360397
BQ.1	EPI_ISL_17848799, EPI_ISL_17848795, EPI_ISL_17848733
BQ.1.1.23	EPI_ISL_17848721, EPI_ISL_16360426
BQ.1.1.15	EPI_ISL_17848728
BQ.1.1.17	EPI_ISL_16360384
BQ.1.1.22	EPI_ISL_17848782
BQ.1.1.24	EPI_ISL_17848725
BQ.1.1.4	EPI_ISL_17848755
BE.9	EPI_ISL_16014612, EPI_ISL_16423443, EPI_ISL_16423450, EPI_ISL_16014613, EPI_ISL_16423451, EPI_ISL_16423452, EPI_ISL_16423453, EPI_ISL_16423454, EPI_ISL_16423455, EPI_ISL_16423456, EPI_ISL_16423457, EPI_ISL_16423458, EPI_ISL_16423444, EPI_ISL_18050262, EPI_ISL_16423446, EPI_ISL_16423448, EPI_ISL_16423449, EPI_ISL_18103824, EPI_ISL_18103822, EPI_ISL_18103821, EPI_ISL_18103823, EPI_ISL_16423435, EPI_ISL_18103825, EPI_ISL_18103826, EPI_ISL_16423436, EPI_ISL_16423438, EPI_ISL_16423439, EPI_ISL_18103827, EPI_ISL_16423437, EPI_ISL_18103828, EPI_ISL_18103833, EPI_ISL_16423440, EPI_ISL_16423441, EPI_ISL_18050264, EPI_ISL_18103829, EPI_ISL_18103831, EPI_ISL_18103835, EPI_ISL_18103830, EPI_ISL_18103832, EPI_ISL_18103836, EPI_ISL_18103837, EPI_ISL_18103834, EPI_ISL_18103816, EPI_ISL_18103815, EPI_ISL_18103817, EPI_ISL_18103820, EPI_ISL_18103819, EPI_ISL_18103842, EPI_ISL_18103843, EPI_ISL_18103838, EPI_ISL_18103839, EPI_ISL_18103840,
BA.5.3.1	EPI_ISL_16360433, EPI_ISL_17848723, EPI_ISL_16360406, EPI_ISL_16423434, EPI_ISL_16360367, EPI_ISL_16423442
BA.5	EPI_ISL_16014611, EPI_ISL_16360424, EPI_ISL_17651888, EPI_ISL_17848760,
BA.5.2.1	EPI_ISL_15803849, EPI_ISL_16002023, EPI_ISL_16002009
BA.5.1.27	EPI_ISL_16423447, EPI_ISL_17848719
BE.10	EPI_ISL_15803842, EPI_ISL_15803843, EPI_ISL_15803845, EPI_ISL_15803848, EPI_ISL_15803852, EPI_ISL_16002010, EPI_ISL_16002011, EPI_ISL_16360390,

Lineage	GISAID accession number
	EPI_ISL_16360392, EPI_ISL_17848720, EPI_ISL_17848767, EPI_ISL_17848783, EPI_ISL_17848802, EPI_ISL_17848732
CK.1	EPI_ISL_15803850, EPI_ISL_16360400, EPI_ISL_16360364, EPI_ISL_18050263, EPI_ISL_16360378, EPI_ISL_17848784, EPI_ISL_17848749, EPI_ISL_17848745, EPI_ISL_17848735, EPI_ISL_17848801, EPI_ISL_17848796,
DL.1	EPI_ISL_16002018, EPI_ISL_16002016, EPI_ISL_16360368
XBB.1	EPI_ISL_16360427, EPI_ISL_17848763, EPI_ISL_17848788
XBB.1.5	EPI_ISL_16706637
BA.4.6	EPI_ISL_16002013, EPI_ISL_16002020, EPI_ISL_17848756
BN.1.3.1	EPI_ISL_16360417, EPI_ISL_17848759
BN.1.5	EPI_ISL_17848747

Appendix Table 2. Characteristics of healthcare workers and inpatients, Hospital Moinhos de Vento, Brazil, November 2022–January 2023

Characteristic	**Total N (%)**	BQ.1	BE.9	BA.5	Other lineages	*p*-value
Total	**272 (100)**	**167 (61.4)**	**52 (19.1)**	**15 (5.5)**	**38 (14.0)**	na
Inpatients	**90 (33.1)**	63 (37.7)	11 (21.2)	4 (26.7)	12 (31.6)	0.152
Healthcare workers	**182 (66.9)**	104 (62.3)	41 (78.8)	11 (73.3)	26 (68.4)	na
Female	**195 (71.7)**	113 (67.7)	46 (88.5)	11 (73.3)	25 (65.8)	0.027
Median age, y (IQR)	**40.4 (31.0–64.4)**	42.6 (32.0–72.2)	34.4 (27.5–43.4)	42.9 (34.0–54.9)	40.8 (28.8–62.4)	0.017*
Previous infection (Yes)	**140 (51.5)**	85 (50.9)	25 (48.1)	9 (60.0)	21 (55.3)	0.821
Symptoms Description†						0.193
Asymptomatic	**17 (6.3)**	15 (9.0)	1 (1.9)	0	1 (2.6)	na
Mild illness	**222 (81.6)**	126 (75.4)	48 (92.3)	14 (93.3)	34 (89.5)	na
Moderate to Severe Illness	**32 (11.8)**	25 (15.0)	3 (5.8)	1 (6.7)	3 (7.9)	na
Unknown	**1 (0.4)**	1 (0.6)	0	0	0	na
Vaccination						0.097
2 Doses	**18 (6.6)**	12 (7.2)	4 (7.7)	0	2 (5.3)	na
3 Doses	**76 (27.9)**	51 (30.5)	12 (23.1)	2 (13.3)	11 (28.9)	na
4 Doses	**176 (64.7)**	104 (62.3)	36 (69.2)	12 (80.0)	24 (63.2)	na
Not vaccinated	**2 (0.7)**	0	0	1 (6.7)	1 (2.6)	na
COVID-19 hospitalization	**36 (13.2)**	25 (15.0)	3 (5.8)	2 (13.3)	6 (15.8)	0.365
Link to known COVID-19 cases	**78 (28.7)**	39 (23.4)	28 (53.8)	5 (33.3)	6 (15.8)	<0.001

BQ.1.1, BQ.1.1.4, BQ.1.1.15, BQ.1.1.17, BQ.1.1.18, BQ.1.1.23, BQ.1.1.24 and BQ.1.3 are aggregated with BQ.1. BA.5.1.27, BA.5.2.1, BA.5.3.1 are aggregated with BA.5. Other lineages include BE.10, CK.1, DL.1, XBB.1, XBB.1.5, BA.4.6, BN.1.3.1, BN.1.5.

*Kruskal-Wallis test was used for continuous variables, the results are presented as median with interquartile ranges [IQR]. p ≤ 0.050 was considered statistically significant.

†Classification According to Clinical Spectrum of SARS-CoV-2 Infection - National Institutes of Health. Chi-square test with adjusted residual analysis was used for categorical variables.

na, not applicable.

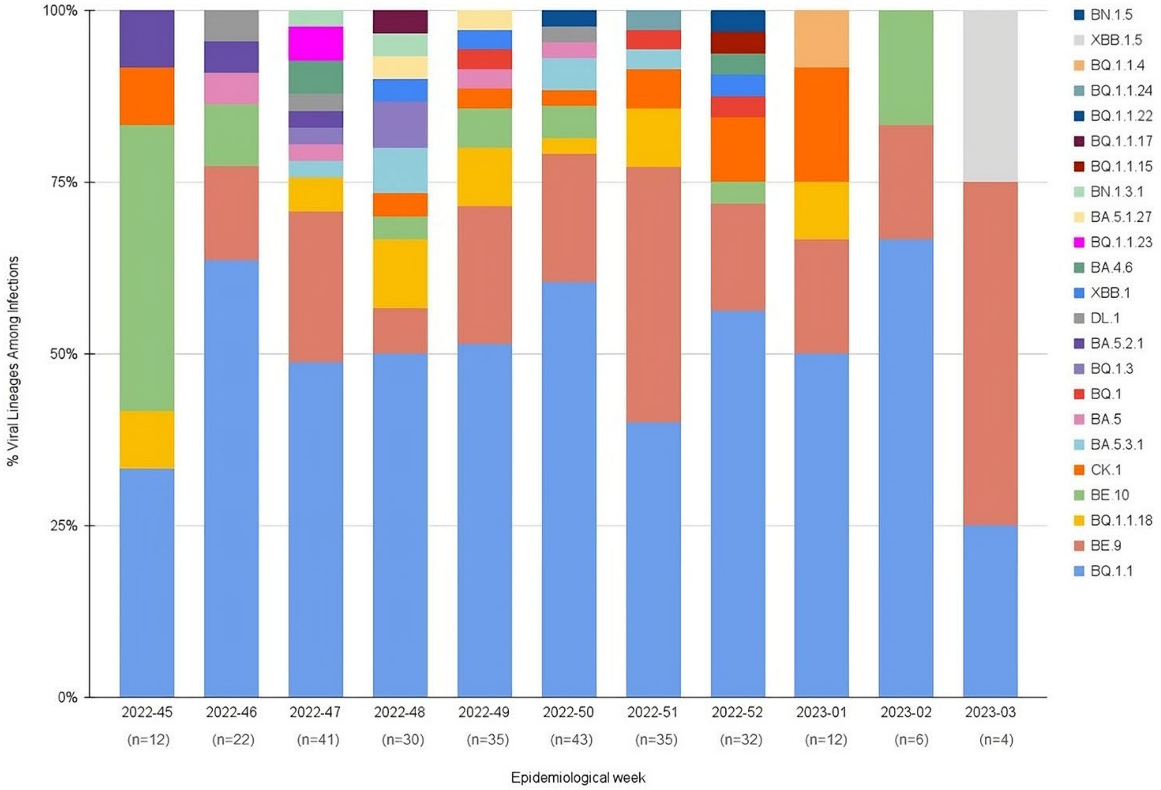

Appendix Figure. Distribution of Omicron SARS-CoV-2 lineages among healthcare workers and inpatients at Hospital Moinhos de Vento in Porto Alegre, southern Brazil, November 2022–January 2023 (n = 272 specimens).
